# Supplementary material for: Changes in Lake Sturgeon Gut Microbiomes Relative to Founding Origin and in Response to Chemotherapeutant Treatments
Source: Microorganisms. 2022 May 10;10(5):1005. doi: 10.3390/microorganisms10051005 (PMC9144364; doi:10.3390/microorganisms10051005)
Supplement: Supplementary file 1 [file microorganisms-10-01005-s001.zip › Table S2.pdf]

Supplementary Table S2. Results from Generalized Linear Models (GLM) in measures of microbial community diversity (a) Inverse Simpson; (b) OTU richness among samples treated with different chemotherapeutants (control group, chloramineT, peroxide, and salt) and from different origins (hatchery and wild eggs).

(a) Inverse Simpson

Model: Inverse Simpson ~ Treatment + Origin

Coefficients	Estimate	Standard Error	t-value	Pr (>t)
**Intercept**	0.278	0.057	4.873	< 0.001***
**Treatments (T) ChloramineT**	-0.024	0.071	-0.335	0.704
**Treatments (T) Peroxide**	-0.038	0.069	-0.554	0.851
**Treatments (T) Salt**	-0.085	0.063	-1.343	0.181
**Origin (O) wild**	-0.008	0.044	-0.182	0.856

Null deviance: 73.277, df 143
Residual deviance: 70.417, df 139
Dispersion parameter for Gamma is 1.335

(b) OTUs richness

Model: OTUs richness ~ Treatment + Origin

Coefficients	Estimate	Standard Error	t-value	Pr (>t)
**Intercept**	5.113	0.144	35.448	< 0.001***
**Treatments (T) ChloramineT**	0.221	0.179	1.235	0.219
**Treatments (T) Peroxide**	0.472	0.172	2.749	<0.01**
**Treatments (T) Salt**	0.725	0.163	4.463	< 0.001***
**Origin (O) wild**	-0.069	0.110	-0.628	0.531

Null deviance: 14058, df 143
Residual deviance: 11517, df 139
Dispersion parameter for Quasipoisson is 103.931
